# Genetic Diversity, Population Structure, and Heritability of Fruit Traits in *Capsicum annuum*

**DOI:** 10.1371/journal.pone.0156969

**Published:** 2016-07-14

**Authors:** Rachel P. Naegele, Jenna Mitchell, Mary K. Hausbeck

**Affiliations:** 1 USDA, Agricultural Research Service, San Joaquin Valley Agricultural Sciences Center, 9611 South Riverbend Avenue, Parlier, CA, United States of America; 2 Department of Plant, Soil and Microbial Sciences, Michigan State University East Lansing, MI 48824, United States of America; Aristotle University of Thessaloniki, GREECE

## Abstract

Cultivated pepper (*Capsicum annuum*) is a phenotypically diverse species grown throughout the world. Wild and landrace peppers are typically small-fruited and pungent, but contain many important traits such as insect and disease resistance. Cultivated peppers vary dramatically in size, shape, pungency, and color, and often lack resistance traits. Fruit characteristics (e.g. shape and pericarp thickness) are major determinants for cultivar selection, and their association with disease susceptibility can reduce breeding efficacy. This study evaluated a diverse collection of peppers for mature fruit phenotypic traits, correlation among fruit traits and Phytophthora fruit rot resistance, genetic diversity, population structure, and trait broad sense heritability. Significant differences within all fruit phenotype categories were detected among pepper lines. Fruit from Europe had the thickest pericarp, and fruit from Ecuador had the thinnest. For fruit shape index, fruit from Africa had the highest index, while fruit from Europe had the lowest. Five genetic clusters were detected in the pepper population and were significantly associated with fruit thickness, end shape, and fruit shape index. The genetic differentiation between clusters ranged from little to very great differentiation when grouped by the predefined categories. Broad sense heritability for fruit traits ranged from 0.56 (shoulder height) to 0.98 (pericarp thickness). When correlations among fruit phenotypes and fruit disease were evaluated, fruit shape index was negatively correlated with pericarp thickness, and positively correlated with fruit perimeter. Pepper fruit pericarp, perimeter, and width had a slight positive correlation with Phytophthora fruit rot, whereas fruit shape index had a slight negative correlation.

## Introduction

Peppers (*Capsicum annuum*) are an important spice and vegetable crop grown in the U.S. and worldwide. In 2014, the U.S. imported $1.6 billion and produced over $834 million of bell and chile peppers [ERS, 2014]. According to the FAO, in 2013 nearly 200 million and 33 million tonnes of green and dry peppers, respectively, were produced worldwide. These numbers include chile, bell, and specialty-type peppers. In the U.S., bell peppers account for $618 million of the pepper market, with chile peppers making up an additional $216 million [NASS, 2014]. Specialty peppers, including cheese-type peppers and those with diverse shape, color or flavor, are a relatively small component of the market. While the U.S. grows predominantly thick walled bell-type peppers, pepper fruit shape can vary greatly [1]. Fruit shape and pericarp or fruit thickness are two of the most important characteristics in deciding a pepper cultivar's regional success. Bell and cheese (sweet pimento style) type peppers are often mild or non pungent with thick flesh. Bell peppers have a blocky, lobed appearance, while cheese peppers are lobed and squat or flat. Pungent peppers, including jalapeno, habanero, serrano, poblano, shishito, and thai peppers, can vary greatly in size, shape, pungency level, color, and flesh thickness [2].

Fruit shape has been extensively studied in the Solanaceae including tomato, pepper and eggplant [1,3–11]. In tomato, quantitative trait loci (QTL) contributing to fruit shape have been identified on chromosomes 2, 3, 7, 8, and 10 [1,3,12,13]. In tomato, fruit shape is primarily determined by allelic variation in the *Sun*, *Ovate*, *Fasciated* (FAS), and *Locule Number* (LC) genes [14]. Rodriguez et al., demonstrated that up to 71% of the specific shape variation could be explained by individual alleles of these genes in a diverse collection of 368 wild and cultivated tomatoes [14]. When QTLs from tomato and pepper were compared, fruit weight was highly co-localized between species, and a single fruit shape QTL was co-localized suggesting conserved elements are contributing to one, if not both, of the traits [1,4].

In pepper, previous studies have evaluated the heritability and effect of QTL associated with fruit horticultural characteristics [2,4,9–11,15,16]. Multiple QTLs have been detected on chromosomes 1–4, 8, 10 and 11 for fruit length, width, and the fruit shape ratio (length:width) [4,9,15,17]. Two major fruit QTLs, designated *fs3*.*1* (fruit shape) and *fs10*.*1* (fruit elongation), were mapped in a BC_4_F_2_ population segregating for fruit shape to chromosomes 3 and 10, respectively [9]. These QTLs explained 67 and 44% of the variation for fruit shape and elongation, respectively, observed in the population. Most recently, Vilarinho et al, evaluated the inheritance of fruit traits in relation to pericarp shape, color thickness and total soluble solids [18]. Based on segregation ratios, they determined that the round shape trait was controlled by a single gene. In a serrano by jalapeno recombinant inbred line F_8_ population, Naegele et al. identified five QTLs contributing to fruit shape and one QTL for pericarp thickness on chromosomes 1,2,4,10 and 3, respectively, explaining 4 to 26% of the variation [15]. Tsaballa et al., evaluated the expression of a gene with sequence similarity to the tomato gene *Ovate* and found significant differences between a round and elongated pepper cultivar [16].

In 2012, another QTL analysis determined that fruit mass, length, diameter, shape ratio, and flesh thickness were controlled by two dominant genes with heritability ranging from 38–88% [10]. When evaluating a pepper germplasm collection from the Caribbean, fruit width was highly heritable, and fruit weight and width were positively correlated, consistent with the QTL analysis by Chaim et al [2,9]. In another mapping study, it was estimated that the heritability of fruit shape and flesh thickness were both 80% [11]. The INRA described the phenotype of over 1,300 pepper accessions in their collection for 12 fruit traits; shape and color were diverse among the domesticated species, while wild species typically had small, elongated fruit [19]. Despite the number of studies evaluating fruit shape in pepper, a limitation to all was the use of subjective visual (elongate, triangular, square, heart, etc.) or manual (length/width ratio) measurements to classify fruit shape. Objective and accurate measurements of fruit will aid in our understanding of the factors of controlling fruit traits. In tomato, improved phenotyping software has been developed, allowing for more objective accurate measurements of fruit characteristics [6,20]. This software has already been successfully implemented in related species [5,6].

While fruit shape is one of the most important considerations for a cultivar, disease resistance is also necessary. Due to breeding bottlenecks, cultivated varieties often do not have resistance to many diseases. Frequently, resistance is identified in small-fruited wild species and incorporated into larger-fruited commercial cultivars [21,22]. Negative horticultural traits may also be transferred along with the positive traits such as disease resistance through linkage drag or as pleiotropic effects. Recently, in tomato, a study demonstrated that undesirable effects on maturity, fruit size, yield and plant architecture were linked to resistance to the late blight pathogen (*Phytophthora infestans*) [23]. In pepper, an overlap between fruit characteristics and disease resistance was identified for a single isolate of *P*. *capsici*, a devastating pathogen that incites fruit, foliar, and root rot [15]. In eggplant, fruit shape was positively correlated with disease susceptibility to *P*. *capsici* in a germplasm population [24]. In kiwi, negative correlations between resistance to the bacterial pathogen *Pseudomonas syringae* pv. *actinidiae* and number of fruit per vine suggested that resistance could result in reduced yield [25]. When transferring disease resistance into commercial cultivars, it is important to identify potential correlations, linkage drag, and pleiotropic effects.

Understanding the heritability, correlation, and diversity of fruit traits is essential for the efficient utilization of pepper germplasm. The objectives of this study were to i) determine fruit horticultural characteristics using the Tomato Analyzer (TA) software, ii) determine population structure associated with fruit traits of interest, iii) associate fruit shape categories with TA values, iv) determine the broad sense heritability for each fruit trait, and v) identify correlations among fruit traits and disease resistance to *Phytophthora capsici*.

## Materials and Methods

One hundred sixteen peppers (*Capsicum annuum*), 114 of which had been previously evaluated for Phytophthora fruit rot resistance, were used in this study (Table 1) [26,27]. Twenty seeds from each line were planted into a 72-cell tray (Hummert Intl.) filled with a soilless-based mix (Suremix, Growers Products Inc. Galesburg, MI) in a polyethylene greenhouse at Michigan State University's Horticulture Research and Teaching Farm (Holt, MI). Seedlings were transferred to 1 L black plastic pots (Hummert Intl.) filled with the same soilless-based mix and grown to maturity. Mature fruit were harvested from each plant, bulked by line, and returned to the lab for evaluation.

**Table 1:** Pepper lines evaluated for fruit characteristics.

Identifier	Country	Continent	Species	Peri^A^	Perim^B^	Area^C^	FSI^D^	M_w^E^	M_h^F^	Fruit Shape	End	Bosland Shape^G^
CM334	Mexico	N. America	*C*. *annuum*	0.11	-	-	-	-	-	Oxheart	Point	Conic
Grif 9094	Greece	Europe	*C*. *annuum*	0.42	11.12	5.78	0.70	3.51	2.44	Rectangular	Blunt	Bell
Grif 9105	Soviet	Asia	*C*. *annuum*	0.35	11.05	6.59	1.23	2.84	3.45	Rectangular	Point	-
Jn566	USA	N. America	*C*. *annuum*	0.37	18.18	15.58	1.07	4.61	4.84	Rectangular	Blunt	-
Jn571	USA	N. America	*C*. *annuum*	0.35	18.33	18.52	1.52	4.11	6.12	Rectangular	Point	-
Jn574	USA	N. America	*C*. *annuum*	0.25	-	-	-	-	-	Oxheart	Point	-
PI 102883	China	Asia	*C*. *annuum*	0.01	-	-	-	-	-	Long	Point	Elongate
PI 123469	India	Asia	*C*. *annuum*	0.09	8.36	3.28	2.76	1.28	3.34	Rectangular	Blunt	Elongate
PI 123474	India	Asia	*C*. *annuum*	0.07	16.10	7.99	3.30	2.04	6.68	Long	Point	Elongate/mixed
PI 124078	India	Asia	*C*. *annuum*	0.08	4.49	1.02	2.06	0.82	1.68	Long	Point	Elongate
PI 135822	Afghanistan	Asia	*C*. *annuum*	0.07	11.65	5.79	2.90	1.65	4.74	Flat	Blunt	Oblate
PI 138557	Iran	Asia	*C*. *annuum*	0.11	10.02	5.59	2.04	1.85	3.78	Rectangular	Point	Conic
PI 138558	Iran	Asia	*C*. *annuum*	0.14	12.07	7.88	1.73	2.39	4.22	Rectangular	Blunt	Conic
PI 138560	Iran	Asia	*C*. *annuum*	0.08	9.06	3.50	2.62	1.40	3.55	Rectangular	Blunt	Conic
PI 138565	Iran	Asia	*C*. *annuum*	0.17	5.60	1.99	1.50	1.33	1.92	Mixed	Blunt	Round
PI 142832	Iran	Asia	*C*. *annuum*	0.10	-	-	-	-	-	Rectangular	Blunt	-
PI 159256	USA	N. America	*C*. *annuum*	-	12.94	6.73	3.45	1.56	5.37	Rectangular	Blunt	-
PI 164311	India	Asia	*C*. *annuum*	0.12	16.44	14.55	2.72	2.48	6.64	Obovoid	Blunt	Elongate
PI 167063	Turkey	Europe	*C*. *annuum*	0.12	13.08	8.96	0.81	4.17	3.03	Mixed	Blunt	Conic
PI 169129	Turkey	Europe	*C*. *annuum*	0.12	11.10	4.97	2.72	1.65	4.42	Long	Point	Elongate
PI 177301	Italy	Europe	*C*. *annuum*	0.12	11.31	5.01	3.11	1.53	4.57	Long	Point	Conic/Mixed
PI 181733	Lebanon	Asia	*C*. *annuum*	0.17	18.39	15.26	1.47	4.13	5.28	Rectangular	Blunt	Elongate/mixed
PI 181734	Lebanon	Asia	*C*. *annuum*	0.18	12.01	7.18	1.84	2.32	4.12	Mixed	Blunt/Point	Elongate/mixed
PI 183922	India	Asia	*C*. *annuum*	0.09	17.74	7.93	3.29	2.37	7.28	Long	Point	Elongate
PI 184039	Serbia	Europe	*C*. *annuum*	0.22	15.36	12.35	2.05	2.93	5.54	Long	Point	Conic/Mixed
PI 201232	Mexico	N. America	*C*. *annuum*	0.18	14.77	5.82	3.37	1.93	5.98	Long	Point	Conic
PI 201234	Mexico	N. America	*C*. *annuum*	0.03	6.84	2.13	2.48	1.23	2.63	Rectangular	Blunt/Point	Elongate
PI 201239	Mexico	N. America	*C*. *annuum*	0.11	13.58	9.94	1.75	2.94	4.85	Long	Point	Conic/Elongate
PI 203524	Cuba	S. America	*C*. *annuum*	0.10	19.14	13.97	3.09	2.63	7.86	Mixed	Blunt	Conic/Mixed
PI 206950	Turkey	Europe	*C*. *annuum*	0.13	19.61	22.95	1.65	4.22	6.82	Rectangular	Blunt	Conic/Mixed
PI 213915	Bolivia	S. America	*C*. *annuum*	0.06	10.09	4.66	2.05	1.80	3.73	Mixed	Point	Elongate/mixed
PI 224438	Mexico	N. America	*C*. *annuum*	0.09	5.47	1.15	3.48	0.69	2.36	Long	Point	Elongate
PI 226633	Iran	Asia	*C*. *annuum*	0.15	10.74	5.99	2.90	1.62	4.30	Rectangular	Point	Elongate
PI 241641	Colombia	S. America	*C*. *annuum*	0.06	13.62	9.18	2.63	2.12	5.37	Rectangular	Blunt	Elongate/mixed
PI 249908	Portugal	Europe	*C*. *annuum*	0.39	17.34	17.68	1.40	4.30	5.71	Oxheart	Point	Conic
PI 250141	Pakistan	Asia	*C*. *annuum*	0.05	7.66	2.86	2.32	1.32	3.04	Long	Point	Elongate/mixed
PI 257047	Colombia	S. America	*C*. *annuum*	0.06	14.96	13.36	1.22	3.94	4.65	Rectangular	Point	Elongate
PI 257048	Colombia	S. America	*C*. *annuum*	0.08	12.14	5.09	2.85	1.75	4.86	Mixed	Point	Elongate
PI 257283	Spain	Europe	*C*. *annuum*	0.24	12.21	8.63	1.61	2.63	4.22	Oxheart	Blunt	Round
PI 263075	Soviet	Asia	*C*. *annuum*	0.13	12.85	6.12	3.00	1.76	5.19	Long	Point	Elongate
PI 263076	Soviet	Asia	*C*. *annuum*	0.18	4.93	1.52	1.35	1.27	1.53	Heart	Point	Elongate/mixed
PI 263077	Soviet	Asia	*C*. *annuum*	0.21	13.58	8.81	2.26	2.30	5.04	Mixed	Point	Conic/Oblate
PI 263113	Soviet	Asia	*C*. *annuum*	0.11	6.07	2.12	1.39	1.44	1.99	Heart	Point	Elongate/mixed
PI 263114	Soviet	Asia	*C*. *annuum*	0.11	6.18	2.19	1.60	1.37	2.01	Rectangular	Blunt	Conic/Elongate
PI 264662	Germany	Europe	*C*. *annuum*	0.20	12.75	6.15	2.26	2.03	4.54	Mixed	Point	Bell
PI 267730	Cuba	S. America	*C*. *annuum*	0.01	2.74	0.52	1.17	0.76	0.88	Rectangular	Point	Conic/Mixed
PI 273415	Italy	Europe	*C*. *annuum*	0.12	11.98	4.42	2.83	1.81	4.52	Long	Point	Elongate
PI 281341	El Salvador	S. America	*C*. *annuum*	0.05	-	-	-	-	-	Long	Point	Mixed
PI 281433	USA	N. America	*C*. *annuum*	0.08	-	-	-	-	-	Rectangular	Blunt	Conic/Mixed
PI 298647	Spain	Europe	*C*. *annuum*	0.22	6.86	2.97	0.95	2.04	1.93	Flat	Blunt	Oblate/Bell
PI 302987	Canada	N. America	*C*. *annuum*	0.21	15.33	7.90	2.31	2.51	5.56	Oxheart	Point	Elongate
PI 339132	Turkey	Europe	*C*. *annuum*	0.10	-	-	-	-	-	Mixed	Point	Conic/Elongate
PI 342949	USA	N. America	*C*. *annuum*	0.25	-	-	-	-	-	Long	Point	Conic/Mixed
PI 357503	Serbia	Europe	*C*. *annuum*	0.25	15.96	7.74	3.31	2.42	5.91	Long	Point	Elongate
PI 357531	Serbia	Europe	*C*. *annuum*	0.29	7.20	3.11	0.85	2.24	1.87	Mixed	Blunt	Bell/Mixed
PI 368396	Serbia	Europe	*C*. *annuum*	0.24	18.77	8.34	2.14	3.62	6.08	Long	Point	Elongate
PI 369996	India	Asia	*C*. *annuum*	0.13	9.23	3.22	2.79	1.32	3.60	Mixed	Point	Elongate
PI 371867	USA	N. America	*C*. *annuum*	0.24	7.76	3.70	1.54	1.73	2.58	Oxheart	Blunt	Elongate
PI 385960	Kenya	Africa	*C*. *annuum*	0.42	8.95	3.88	0.85	2.69	2.26	Mixed	Blunt	Bell
PI 409141	South Africa	Africa	*C*. *annuum*	0.03	11.26	5.45	2.91	1.59	4.62	Long	Point	Conic
PI 410407	Brazil	S. America	*C*. *annuum*	0.25	7.78	3.23	1.02	2.15	2.13	Rectangular	Point	Conic
PI 427290	USA	N. America	*C*. *annuum*	0.10	19.06	8.04	4.28	1.88	7.98	Long	Point	Conic
PI 432802	China	Asia	*C*. *annuum*	0.51	13.17	9.81	0.82	3.82	3.19	Mixed	Blunt	Bell
PI 438624	Mexico	N. America	*C*. *annuum*	0.06	5.61	2.12	1.37	1.43	1.83	Mixed	Blunt	Conic
PI 438633	Mexico	N. America	*C*. *annuum*	0.09	10.14	4.31	2.34	1.64	3.83	Rectangular	Point	Elongate
PI 441628	Brazil	S. America	*C*. *annuum*	0.10	10.46	6.83	1.94	2.04	3.87	Rectangular	Point	Conic
PI 511879	Mexico	N. America	*C*. *annuum*	0.08	16.45	8.45	4.37	1.55	6.98	Long	Point	Elongate
PI 511882	Mexico	N. America	*C*. *annuum*	0.16	14.27	10.09	1.92	2.84	5.03	Long	Point	Conic/Elongate
PI 511884	Mexico	N. America	*C*. *annuum*	0.08	5.93	2.27	1.93	1.15	2.16	Mixed	Point	Conic
PI 550700	USA	N. America	*C*. *annuum*	0.19	18.52	12.13	2.22	2.79	6.52	Mixed	Point	-
PI 566808	Mexico	N. America	*C*. *annuum*	0.32	15.28	12.13	1.66	3.19	5.20	Mixed	Blunt/Point	-
PI 566811	Mexico	N. America	*C*. *annuum*	0.15	-	-	-	-	-	Long	Point	Elongate
PI 585246	Ecuador	S. America	*C*. *annuum*	0.06	11.85	6.82	2.38	1.91	4.60	Long	Point	Elongate
PI 593493	Mexico	N. America	*C*. *annuum*	0.02	2.89	0.50	1.76	0.63	1.10	Rectangular	Point	-
PI 593495	Mexico	N. America	*C*. *annuum*	0.01	5.07	1.37	2.13	0.95	1.96	Ellipsoid	Blunt	Conic
PI 593511	Mexico	N. America	*C*. *annuum*	0.07	10.28	4.29	3.44	1.29	4.31	Long	Point	-
PI 593561	USA	N. America	*C*. *annuum*	0.18	5.29	1.72	1.22	1.44	1.75	Rectangular	Point	Elongate
PI 593564	Mexico	N. America	*C*. *annuum*	0.04	-	-	-	-	-	Rectangular	Blunt	Elongate
PI 593573	Brazil	S. America	*C*. *annuum*	0.12	6.75	2.07	2.64	1.03	2.68	Oxheart	Point	Conic
PI 593920	Ecuador	S. America	*C*. *frutescens*	0.05	12.85	7.79	2.65	1.98	5.21	Long	Point	Elongate
PI 593929	Venezuela	S. America	*C*. *chinense*	0.23	6.40	2.24	0.79	2.10	1.65	Mixed	Point	Round
PI 593933	Ecuador	S. America	*C*. *annuum*	0.06	4.69	1.40	1.18	1.27	1.48	Heart	Point	Campanulate
PI 595906	Venezuela	S. America	*C*. *annuum*	0.06	4.18	1.02	1.67	0.90	1.51	Mixed	Blunt	Mixed
PI 600934	USA	N. America	*C*. *annuum*	0.12	23.53	11.60	2.87	3.57	8.80	Long	Point	-
PI 601110	USA	N. America	*C*. *annuum*	0.39	-	-	-	-	-	Rectangular	Blunt	-
PI 631126	China	Asia	*C*. *annuum*	0.33	-	-	-	-	-	Flat	Blunt/Point	Bell
PI 631131	Yemen	Asia	*C*. *annuum*	0.13	-	-	-	-	-	Long	Point	Elongate
PI 631140	Guatemala	N. America	*C*. *annuum*	0.13	5.12	1.53	1.79	1.05	1.88	Oxheart	Point	Elongate
PI 631143	Guatemala	N. America	*C*. *annuum*	0.06	7.79	3.01	0.85	2.17	1.85	Round	Blunt	Conic
PI 631147	India	Asia	*C*. *annuum*	0.09	-	-	-	-	-	Rectangular	Point	Elongate
PI 639641	Poland	Europe	*C*. *annuum*	0.37	13.47	7.54	1.24	3.22	3.83	Rectangular	Blunt	Bell
PI 640448	Taiwan	Asia	*C*. *annuum*	0.12	13.81	3.78	4.10	1.40	5.53	Long	Point	-
PI 640460	China	Asia	*C*. *annuum*	0.23	8.32	3.81	1.35	2.00	2.60	Rectangular	Blunt	-
PI 640461	China	Asia	*C*. *annuum*	0.09	4.72	0.99	2.93	0.68	1.97	Long	Point	-
PI 640480	France	Europe	*C*. *annuum*	0.17	7.26	2.99	1.00	2.06	2.06	Mixed	Point/Blunt	-
PI 640516	Taiwan	Asia	*C*. *annuum*	0.09	-	-	-	-	-	Round	Blunt	-
PI 640532	Mexico	N. America	*C*. *annuum*	0.33	12.81	8.90	1.45	2.88	4.14	Oxheart	Point	-
PI 640579	Egypt	Africa	*C*. *annuum*	0.11	-	-	-	-	-	Long	Point	-
PI 640581	Nigeria	Africa	*C*. *annuum*	0.05	-	-	-	-	-	Rectangular	Blunt	-
PI 640582	Nigeria	Africa	*C*. *annuum*	0.09	10.09	5.36	2.59	1.52	4.08	Rectangular	Blunt	-
PI 640588	USA	N. America	*C*. *annuum*	0.17	4.13	1.13	1.01	1.16	1.17	Heart	Blunt	-
PI 640641	Indonesia	Asia	*C*. *annuum*	0.04	11.79	3.91	3.11	1.59	4.78	Long	Point	-
PI 640659	Thailand	Asia	*C*. *annuum*	0.10	18.13	11.67	3.87	2.01	7.63	Long	Blunt	-
PI 640663	Taiwan	Asia	*C*. *annuum*	0.10	7.06	1.52	3.49	0.85	2.94	Long	Point	-
PI 640670	India	Asia	*C*. *annuum*	0.03	9.39	3.39	3.29	1.23	4.00	Long	Point	-
PI 640671	Sri Lanka	Asia	*C*. *annuum*	0.04	18.64	13.43	2.95	2.59	7.45	Long	Blunt	
PI 640676	Kenya	Africa	*C*. *annuum*	0.08	16.73	6.52	2.78	2.47	6.16	Rectangular	Blunt/Point	-
PI 640682	Tanzania	Africa	*C*. *annuum*	0.06	6.31	1.83	2.62	1.00	2.51	Long	Point	-
PI 640744	Japan	Asia	*C*. *annuum*	0.10	-	-	-	-	-	Oxheart	Point	-
PI 640791	Egypt	Africa	*C*. *annuum*	0.18	25.53	20.82	3.15	3.16	9.96	Long	Point	-
PI 640803	Philippines	Asia	*C*. *annuum*	-	9.29	2.09	4.29	0.95	3.86	Long	Point	-
PI 640809	Denmark	Europe	*C*. *annuum*	0.06	7.32	1.92	3.56	0.84	3.02	Long	Point	-
PI 640815	Zambia	Africa	*C*. *annuum*	-	17.26	19.84	3.19	2.36	6.84	Obovoid	Blunt	-
PI 640833	USA	N. America	*C*. *annuum*	0.07	6.20	2.38	1.44	1.46	2.11	Heart	Point	-
PI 645520	Italy	Europe	*C*. *annuum*	0.47	23.53	23.26	0.64	7.27	4.62	Mixed	Blunt	-
PI 653650	Bangladesh	Asia	*C*. *annuum*	0.08	-	-	-	-	-	Rectangular	Point	-

^A^ Fruit pericarp thickness (cm).

^B^ Fruit perimeter (cm).

^C^ Fruit area (cm^2^)

^D^ Fruit Shape Index 1 as described by Tomato Analyzer [20].

^E^ Maximum width.

^F^ Maximum height.

^G^ Fruit shape described by Bosland [27].

Clean mature pepper fruit were sliced longitudinally, placed face down on an Epson Perfection V30 scanner (Epson America, Long Beach, CA), and scanned. Using the Tomato Analyzer (TA) software v3.0, fruit perimeter, area, width at mid height, max width, height at mid width, max height, shoulder height and fruit shape index external 1 were determined as described [6,13,20]. Fruit shape categories Circular (smaller values indicate more circular), Rectangular (ratio of the area of the shape containing the fruit to the area of the rectangle contained by the fruit), Ellipsoid (smaller values indicate fruit is more ellipsoid), Ovoid and Obovoid were calculated by TA. When the software was unable to accurately identify the outline of a fruit shape, or proximal or distal ends, points were adjusted manually. Fruit end shape (pointed or blunt) was assessed visually for each line. Fruit shape (Long, Ellipsoid, Rectangular, Oxheart, Heart, Round, Flat) was assessed visually and categorized using the designations described by Rodriguez et al. [14]. Additionally, fruit shape categories (Elongate, Oblate, Round, Conic, Campanulate, Bell, Mixed) for 79 accessions that had previously been characterized by Bosland, were also included [27]. Fruit pericarp thickness was measured using a hand caliper on each side of a longitudinal slice and averaged for each fruit.

Data were analyzed in the software SAS v9.3 (SAS Cary, NC) using the PROC MIXED function. Significant differences were detected using ANOVA and separated using LSD (P = 0.05). For fruit shape index, perimeter, and area, data were natural log transformed to fulfill assumptions of normality. Correlations were detected using Pearson's Correlation coefficient (r) at *P* = 0.05 among fruit traits and disease. Only lines for which complete TA data and disease data were available were used for correlation analyses. Disease data from a previous study were used for lesion area at three and five days post inoculation (dpi) [26]. Only the first two reps (for a total of 10 peppers) were used for fruit characteristics and disease correlations. Broad sense heritability for each trait was estimated using the mean squares implemented within the formula described by Fehr [28]. Confidence intervals were calculated according to Knapp et al. [29].

Previously, twenty-three simple sequence repeat (SSR) markers were evaluated for the population [26]. For the subset of pepper lines evaluated in this study, genetic structure of the population was evaluated in the software STRUCTURE v3.4 [30] with a burn in of 300,000 and a MCMC of 500,000 with correlated allele frequencies [31]. To test the putative number of populations, K values of 1–15 were evaluated with three independent runs. Lambda was estimated at 0.55 and the value of K was reported to be five according to the methods by Evanno et al [32] implemented in STRUCTURE Harvester [33]. The significance of Wright's F_ST_, a measure of the genetic differentiation among sub populations, was determined using PowerMarker v3.25 [34] with 1,000 permutations. Differentiation was defined according to Hartl and Clark [35]. Population structure was sorted by predefined categories (pericarp thickness, fruit shape, and end shape) using the Population Sorting Tool [24]. Lines were considered to belong to a cluster if they had a membership (Q) ≥ 60% in that cluster. For categorical analyses in STRUCTURE, pepper lines were grouped based on a pericarp thickness of <0.05, 0.05 to 0.10, 0.11 to 0.15, 0.16 to 0.20, 0.21 to 0.30, or ≥0.30 cm. Only categories represented by three or more individuals with unmixed fruit were included in population structure and geographic-level ANOVA analyses.

## Results and Discussion

**Fig 1:**
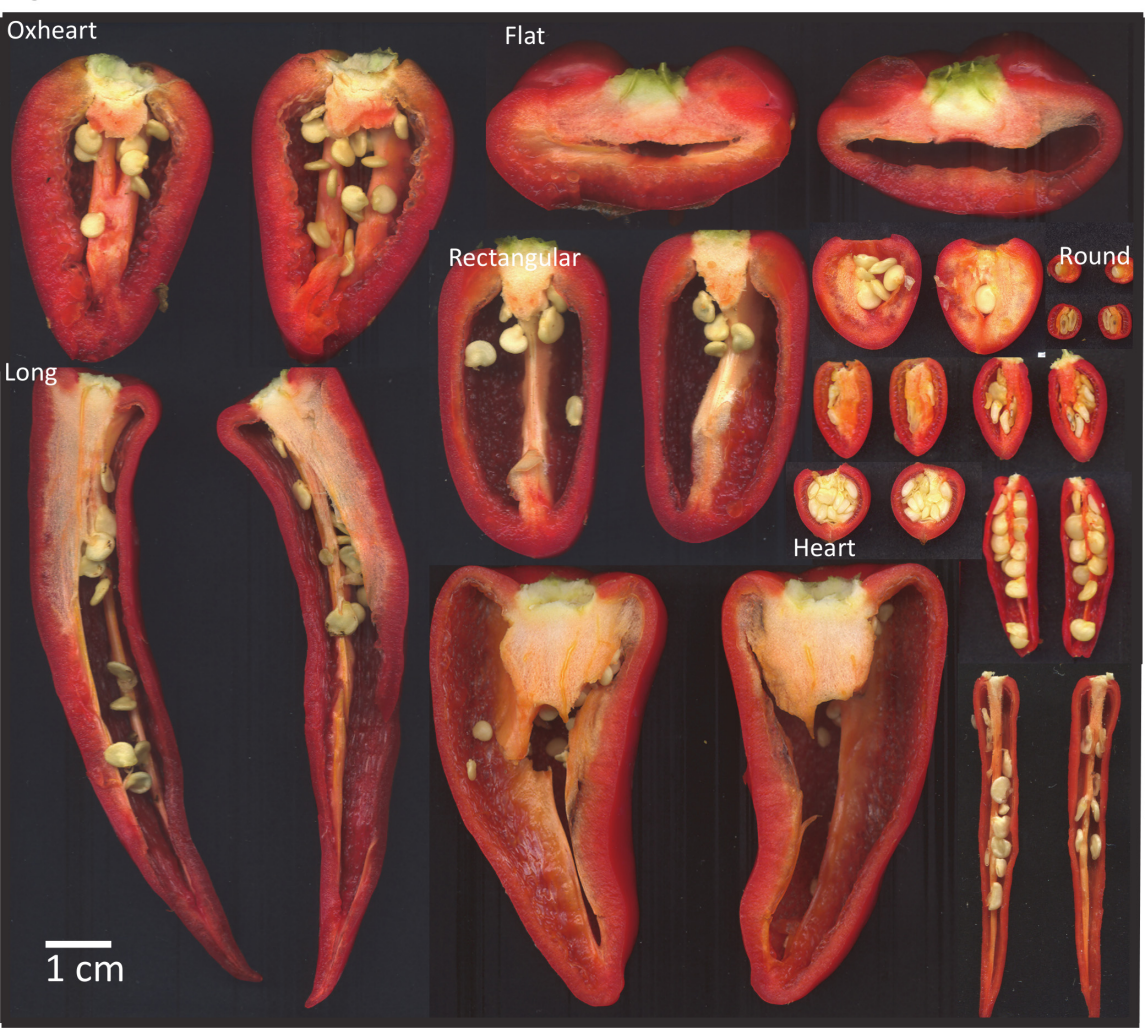
Mature pepper fruit phenotypic diversity in size, shape, end shape, and pericarp thickness of a worldwide collection.

Since their initial domestication in Mexico, peppers have been under strong selection for fruit shapes and size [36]. While wild relatives and landrace peppers are frequently small and highly pungent, domesticated pepper fruit have an endless array of phenotypic diversity [2,19]. For most countries and markets, there are distinct regional preferences for the type of pepper consumed. These regional preferences have contributed to strong phenotypic diversity among market classes [37]. In this study, fruit traits varied in the population, and significant differences were detected among lines for each of the phenotypic traits evaluated (Fig 1, Table 1, S1 Table). The mean pericarp thickness of the population was 0.14 ± 0.002 cm. The lines with the thinnest pericarp were PIs 267730, 593495, and 102883 (0.01 cm). The line with the thickest pericarp was PI 432802 (0.51 cm). When grouped by continent, the pericarp thickness of fruit from Europe was the highest (0.22), while fruit from South America had the thinnest pericarp (0.09). When grouped by country, fruit from Serbia were the thickest (0.25) while fruit from Ecuador were the thinnest (0.06 cm) (Table 2). Many of the accessions from South America were wild or landrace individuals, and had thin fruit compared to the cultivated fruit from Europe, which were more than twice as thick on average. The variation in pericarp thickness was also detected among countries.

**Table 2:** Fruit thickness, perimeter, area and fruit shape compared among countries and continents.

		Thickness (cm)		Perimeter (cm)		Area (cm^2^)		FSI^A^	
**Continents**									
	Europe	0.22	A^**B**^	12.55	A	6.91	A	1.54	C
	N. America	0.15	B	9.47	BC	4.19	BC	2.01	B
	Asia	0.13	C	9.66	B	4.02	C	2.31	A
	Africa	0.13	C	11.69	A	5.14	B	2.40	A
	S. America	0.09	D	8.53	C	3.85	C	1.82	B
**Country**									
	Serbia	0.25	A	13.33	CDE	6.26	DF	1.82	IJKLMN
	China	0.24	AB	7.91	KLMN	3.36	HIKL	1.55	MNOP
	Italy	0.24	ABC	14.56	BCD	7.84	CD	1.91	HIJKLM
	USA	0.21	BC	11.24	EFGH	5.61	F	1.87	IJKL
	Soviet	0.18	DE	8.21	JKLM	3.51	HIK	1.71	JKLMN
	Brazil	0.16	EF	7.84	LMN	3.13	IKL	1.78	IJKLMN
	Turkey	0.12	FGHI	13.76	BCD	9.31	BCE	1.59	LMNO
	Iran	0.12	FGH	8.99	IJKL	4.08	HI	2.08	GHI
	Mexico	0.11	GHI	8.58	JKL	3.54	HIK	2.23	FGH
	Taiwan	0.10	GHIJK	9.63	GHIJKL	2.35	KLM	3.70	AB
	India	0.09	IJK	10.47	FGHI	4.30	GHI	2.83	CD
	Colombia	0.07	JK	13.23	CDE	8.01	BCD	2.09	GHI

^A^ Fruit Shape Index 1 as defined by Tomato Analyzer

^B^ Numbers followed by the same letter within a column are not significantly different at P ≤ 0.05.

The line with the smallest perimeter was PI 267730 (2.74 cm) and the line with the largest perimeter was PI 640791 (25.53 cm). The mean perimeter for the population was 11.39 ± 0.20 cm. Fruit from Italy had the largest perimeter (14.56 cm) and fruit from Brazil had the smallest (7.84 cm). Fruit with the largest perimeter (12.55 cm) came from Europe; fruit from South America had the smaller perimeter (8.52 cm). The population mean for fruit area was 6.71 ± 7.68 cm^2^; the smallest was 0.50 cm^2^ (PI 593493) and the largest was 23.26 cm^2^ (PI 645520). Turkey (9.31 cm^2^) had fruit with the largest area, whereas fruit from Taiwan were the smallest (2.35 cm^2^). Fruit from Europe had the greatest area (6.91 cm^2^) and fruit from South America had the smallest (3.85 cm^2^). PI 645520 (0.64) and PI 511879 (4.37) had the lowest and highest fruit shape index, respectively. The population mean for fruit shape index was 2.19 ± 1.12. Fruit from Africa (2.40) and Taiwan (3.70) had the largest fruit shape index, while fruit from Europe (1.54) and China (1.55) had the lowest. The smallest maximum width and height for the population was 0.63 cm (PI 593495) and 0.88 cm (PI 267730), respectively. The largest maximum width and height for the population was 7.27 cm (PI 645520) and 9.96 cm (PI 640791), respectively. The population means for maximum width and height was 2.13 ± 1.27 cm and 4.11 ± 2.41 cm, respectively. Broad sense heritability was high (>0.90) for most fruit traits evaluated (Table 3). Pericarp had the highest heritability in the population (0.98). Fruit shape index 1 and width at mid height also had high heritability (0.96) in the population. The lowest heritability was observed for shoulder height (0.56). Previous studies have shown that heritability of fruit shape (length to width ratio) and pericarp thickness are high in peppers [2,9–11]. Consistent with previous research, this pepper population had high heritability (>0.90) for most of the traits evaluated. The traits with lowest heritability in the population were shoulder height (0.56) and fruit shape triangle (0.84) suggesting these attributes are more subject to environmental variation.

**Table 3:** Broad sense heritability of fruit phenotypic characteristics.

Trait	Heritability
Perimeter (cm)	0.94
Pericarp (cm)	0.98
Area (cm^2^)	0.88
Width at Mid Height (cm)	0.96
Maximum Width (cm)	0.95
Height at Mid Width (cm)	0.92
Maximum Height (cm)	0.94
Fruit shape index 1	0.96
Fruit shape triangle	0.67
Ellipsoid	0.92
Rectangular	0.84
Circular	0.97
Shoulder Height	0.56

**Fig 2:**
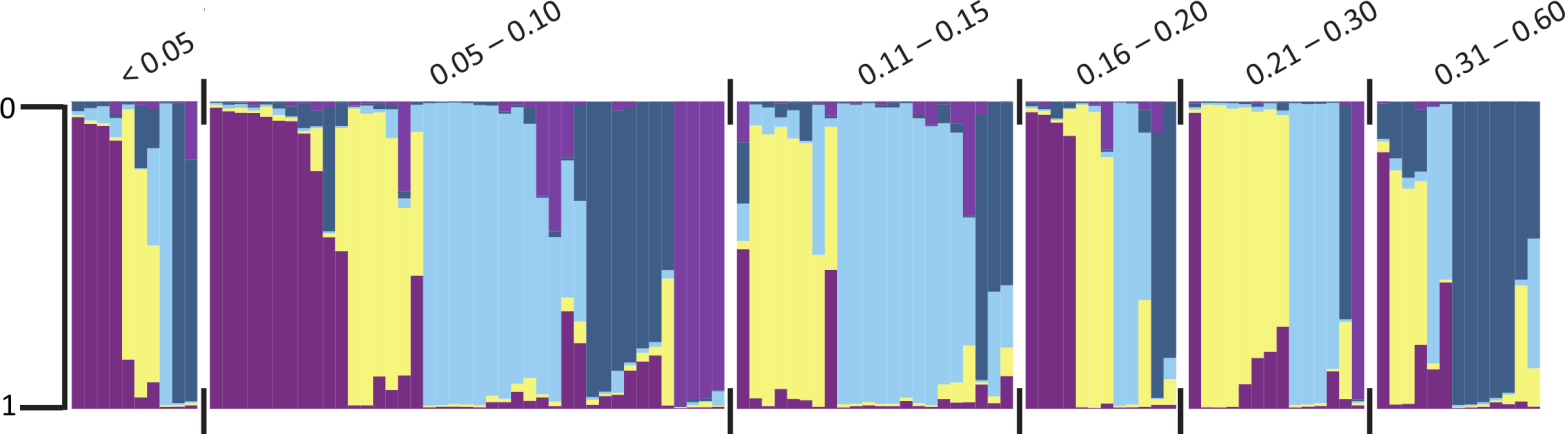
Population structure of pepper (*Capsicum annuum*) grouped by pericarp thickness categories. Individuals are represented by their proportionate membership (0 to 1) in cluster 1 (purple), cluster 2 (light yellow), cluster 3 (sky blue), cluster 4 (steel blue), or cluster 5 (orchid). A white space and black tick marks separate subgroups of individuals.

The software STRUCTURE detected 5 genetic clusters (Ln = -3,526.3). The genetic differentiation between clusters was moderate to very great (F_ST_ = 0.06–0.16). Clusters did not perfectly differentiate fruit shape or pericarp thickness categories. However, certain clusters were more frequently associated with a particular category (Fig 2) than others. When grouped by pericarp thickness, genetic diversity and polymorphism information content (PIC) were moderate among groups (Table 4). The highest PIC and genetic diversity were in fruit from the 0.05–0.10 (PIC = 0.40, GD = 0.44) and 0.16–0.20 (PIC = 0.40, GD = 0.45) categories. When grouped by pericarp thickness, cluster 4 (dark blue) was less frequently found in peppers with a pericarp <0.05, 0.11 to 0.15, and 0.21 to 0.30. Cluster 2 (yellow) was less frequently associated with peppers with a pericarp <0.05 or ≥0.30. Little differentiation (F_ST_ = 0 to 0.05) was detected between peppers with a pericarp thickness of <0.05 or 0.05 to 0.10 and peppers with a pericarp thickness of 0.16 to 0.20, or peppers with a pericarp thickness of 0.16 to 0.20 and peppers with a pericarp thickness ≥0.30 (Table 5). Moderate differentiation (F_ST_ = 0.05 to 0.15) was detected between peppers with a pericarp thickness of 0.05 to 0.10 and peppers with a pericarp thickness ≥0.30. These data, combined with pericarp differences among continents, suggest that the differentiation is a result of pericarp thickness and not just a pleiotropic difference between wild and cultivated lines.

**Table 4:** Genetic diversity of pepper fruit pericarp thickness.

Category	Allele Freq^A^	GenoNo^B^	AlleleNo^C^	GD^D^	Heterozygosity	PIC^E^
**<0.05**	0.71	2.70	2.78	0.40	0.12	0.35
**0.05–0.10**	0.68	4.30	3.70	0.44	0.13	0.40
**0.11–0.15**	0.71	3.48	3.13	0.39	0.17	0.35
**0.16–0.20**	0.65	3.04	2.96	0.45	0.14	0.40
**0.21–0.30**	0.71	2.91	2.87	0.39	0.12	0.34
**0.31–0.60**	0.69	2.74	2.65	0.41	0.11	0.35

^A^Frequency of the major allele.

^B^Number of genotypes.

^C^Number of alleles detected.

^D^Gene Diversity.

^E^Polymorphism information content.

**Table 5:** Genetic differentiation of pepper lines when grouped by pericarp thickness (cm).

Category	0.05–0.10	0.11–0.15	0.16–0.20	0.21–0.30	0.31–0.60
**<0.05**	0.08	0.05	0.05*	0.06	0.05
**0.05–0.10**	-	0.04	0.04*	0.09	0.10*
**0.11–0.15**		-	0.02	0.04	0.07
**0.16–0.20**			-	0.03	0.05*
**0.21–0.30**				-	0.08

*Indicates a significant value at P ≤ 0.05.

**Fig 3:**
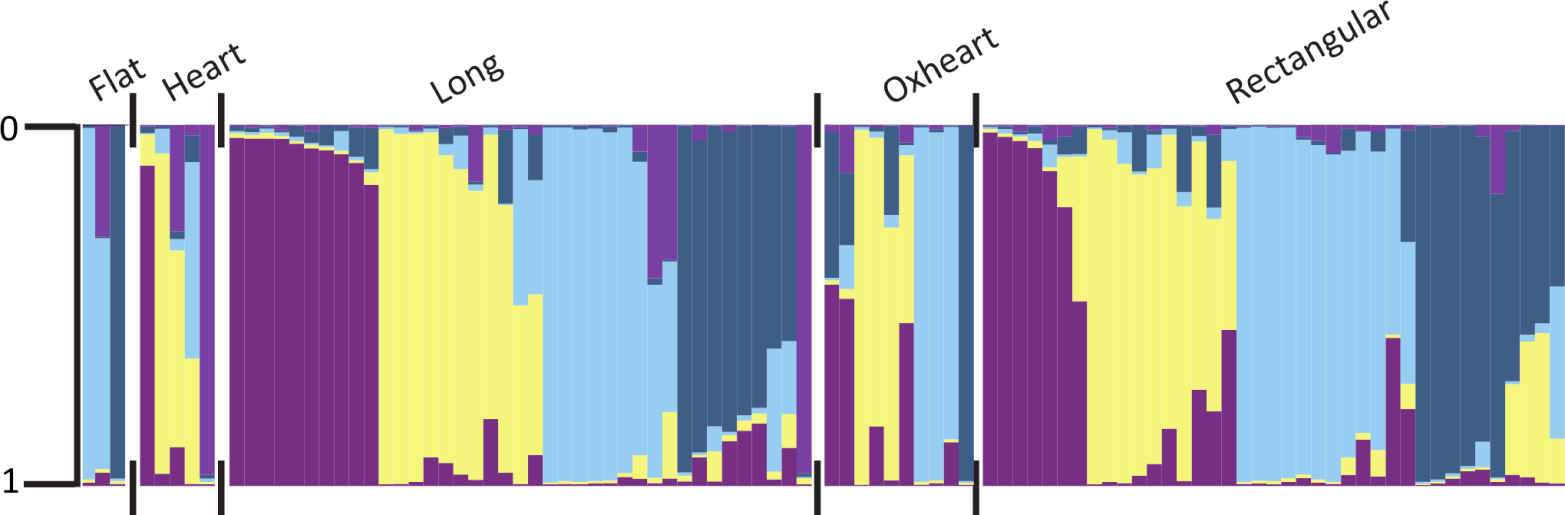
Population structure of pepper (*Capsicum annuum*) grouped by fruit shape categories described by Rodriguez et al [14]. Only categories represented by more than four individuals are included. Individuals are represented by their proportionate membership (0 to 1) in cluster 1 (purple), cluster 2 (light yellow), cluster 3 (sky blue), cluster 4 (steel blue), or cluster 5 (orchid). A white space and black tick marks separate subgroups of individuals.

**Fig 4:**
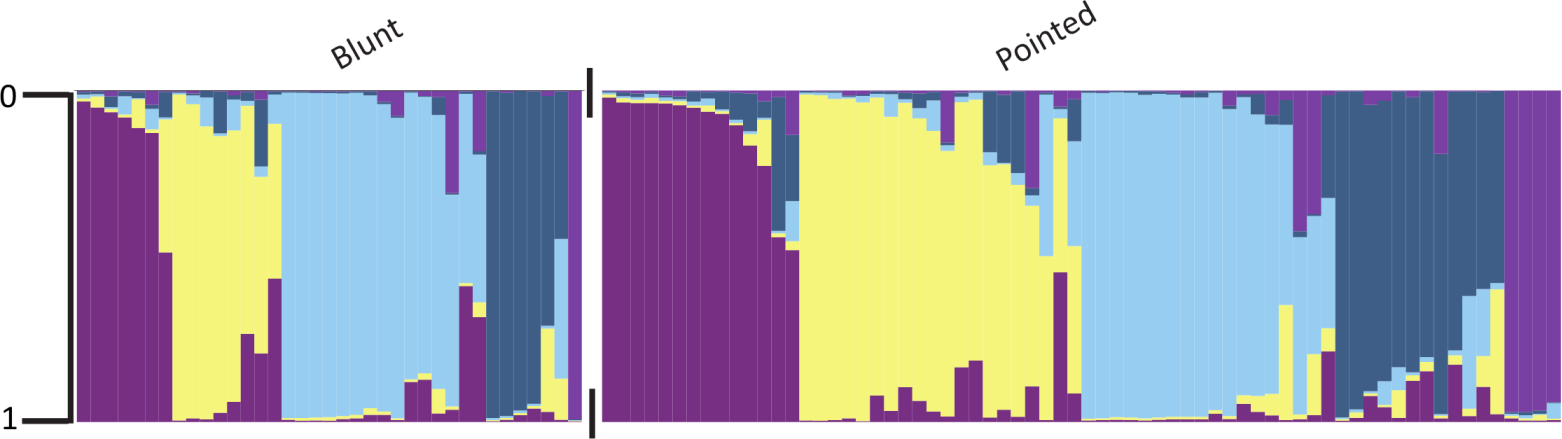
Population structure of pepper (*Capsicum annuum*) grouped by fruit end shape categories. Individuals are represented by their proportionate membership (0 to 1) in cluster 1 (purple), cluster 2 (light yellow), cluster 3 (sky blue), cluster 4 (steel blue), or cluster 5 (orchid). A white space and black tick marks separate subgroups of individuals.

**Fig 5:**
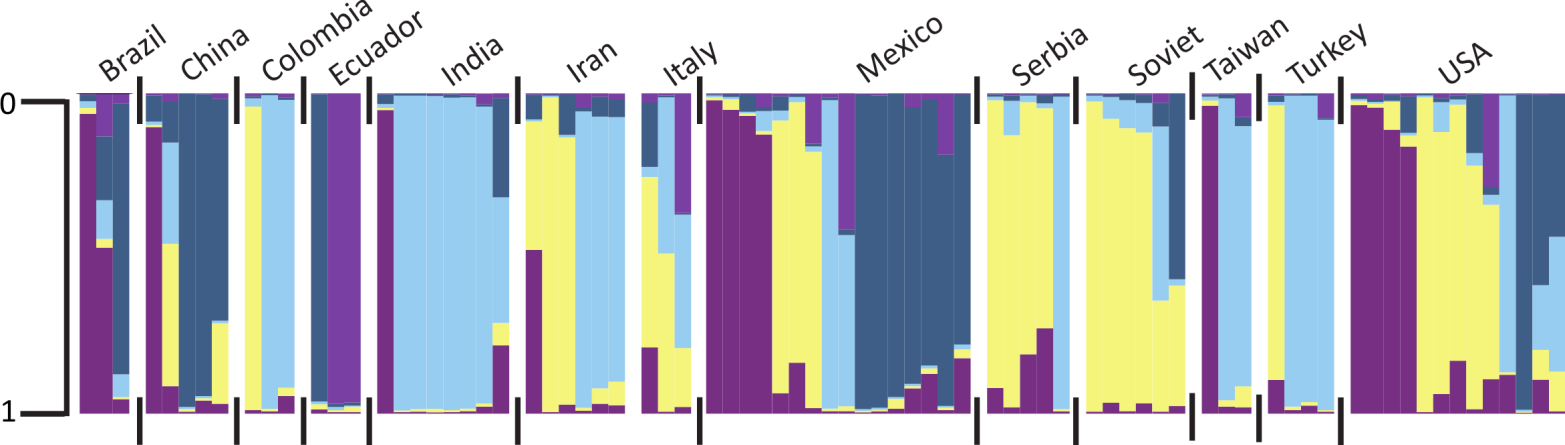
Population structure of pepper (*Capsicum annuum*) grouped by country of origin. Only countries represented by more than four individuals are included. Individuals are represented by their proportionate membership (0 to 1) in cluster 1 (purple), cluster 2 (light yellow), cluster 3 (sky blue), cluster 4 (steel blue), or cluster 5 (orchid). A white space and black tick marks separate subgroups of individuals.

For pepper fruit shape, moderate to very great differentiation was detected among many of the predefined categories using the descriptors designated by Rodriguez et al [14]. Flat peppers were very greatly differentiated from long and rectangular peppers, but not significantly differentiated from oxheart-shaped peppers (Table 6). Oxheart-shaped and rectangular peppers had little differentiation from long peppers. Cluster 3 (light blue) was not detected in the heart shape category. Clusters 1 (dark purple) and 5 (orchid) were not detected in the flat or oxheart categories (Fig 3). Cluster 5 was also not detected in the rectangular category. The round pepper category was not represented by three or more individuals, and comparisons could not be made with remaining fruit shape categories. For the Bosland shape descriptors, no significant differentiation was detected among categories. End shape (pointed or blunt) had little differentiation (0.0001) among the subpopulations. Only cluster 5 (orchid) was underrepresented in blunt individuals (Fig 4). When grouped by country, clusters did not perfectly coincide with categories (Fig 5).

**Table 6:** Genetic differentiation of pepper lines when grouped by fruit shape categories^A^.

Category	Flat	Heart	Long	Obovoid	Oxheart	Rectangular
**Ellipsoid**	0.46*	0.54*	0.87*	0.69*	0.68*	0.87*
**Flat**	-	0.08	0.29*	0.44*	0.15	0.33*
**Heart**		-	0.01	0.54*	0.07	0.07
**Long**			-	0.88*	-0.0009*	0.01*
**Obovoid**				-	0.70*	0.88*
**Oxheart**					-	0.03

^A^ Fruit shape categories described by Rodriguez et al [14].

*Indicates a significant value at P ≤ 0.05.

When fruit shape index parameters from TA were compared to visual fruit shape categories, variation was evident. For heart-shaped fruit, the average fruit shape index was 1.28 and ranged from 1.01 to 1.44. The average maximum width was 1.32 cm and ranged from 1.16 to 1.46 cm and the average maximum height was 1.65 cm with a range of 1.17 to 2.11 cm. For long fruit, the average fruit shape index was 3.06 and ranged from 1.75 to 4.29. The maximum width for long fruit was 1.84 cm with a range of 0.68 to 3.57 cm. The average maximum height was 1.65 cm with a range of 1.17 to 2.11 cm. For rectangular fruit, the average fruit shape index was 1.86 and ranged from 0.70 to 3.45. The average maximum width was 2.31 cm with a range of 0.63 to 4.22 cm, and the average maximum height was 3.78 cm with a range of 0.88 to 6.82 cm. Smaller fruit with a higher fruit shape index were frequently found in South America. North America wasn't significantly different, and could be the result of including small-fruited breeding lines. Population structure also supported differences among categories with certain clusters being more frequently associated with some categories compared to others. For example, cluster 1 (dark purple) was more frequently associated with thinner pericarp, and long or rectangular shaped peppers (Fig 3). This cluster was also more frequently associated with fruit from Mexico and the USA, consistent with similarities in fruit shape index and size. Combining these data with metabolomic or disease data could provide useful tools for germplasm selection [26,38].

The combination of markers and fruit shape categories used in this study was not sufficient for separating the population structure of fruit shape. When fruit shape was designated using the terms described by Bosland, no significant differentiation was detected among categories. When fruit shape was grouped by categories described by Rodriguez [14,39], significant differentiation was detected between five of the category combinations. However, the Rodriguez categories were not sufficient to perfectly differentiate each shape using these markers. Fruit shape index, maximum width, and maximum height values from TA were associated with a range of values for each of the categorical descriptors of shape. For heart and oxheart-shaped peppers, the fruit shape index ranges were small and may be predictive of actual fruit shape. For long and rectangular peppers, however, the fruit shape index ranges were broad suggesting that further division of shape categories will be needed.

Combining subjective definitions such as those employed by the INRA with objective TA measurements may improve separation of shape categories in pepper [19,37]. Using a controlled and accurate categorical definition of fruit shape in pepper will improve the classification and delineation of shape categories, which in turn can improve our ability to determine genetic components controlling shape. Further refinement of fruit shape categories, their associations to fruit shape alleles such as *caOvate* and fruit shape index values are needed to further our understanding of fruit shape in pepper.

The fruit shape index was positively correlated with fruit perimeter (r = 0.2267, *P*<0.0001), and height (midpoint (r = 0.4979, *P*< 0.0001) and maximum (r = 0.4822, *P*<0.0001)), but negatively correlated with fruit pericarp (r = -0.3587, *P*<0.0001) and width (midpoint (r = -0.4626, *P*<0.0001) and maximum (r = -0.3915, *P*<0.0001)). Fruit shape index is measured as the ratio of fruit length to width, and previously studies have shown positive and negative correlations with fruit length and width, respectively [40]. Similarly pericarp thickness was negatively correlated with fruit width, consistent with results from Dwivedi et al [41], while fruit shape index was negatively associated similar to Rao et al [42]. Fruit shape and flesh thickness are important considerations for cultivar classification (bell, cheese, jalapeno, habanero, serrano, poblano, shishito, and thai). Linkage between fruit shape characteristics could affect the speed at which, traits such as flavor compounds could be integrated from chili-type peppers into the sweet bell and cheese-type peppers.

Fruit shape triangle (the ratio of the width at the upper position to the width at the lower position) was positively correlated only with pericarp thickness (r = 0.1233, *P* = 0.0002). Tomato Analyzer fruit shape identifiers (Elliptical, Circular, Rectangular, Obovoid and Ovoid) varied in correlation and significance with remaining fruit categories (S2 Table). Shoulder height was positively correlated with pericarp thickness (r = 0.1541, *P*<0.0001), perimeter (r = 0.0999, *P* = 0.0019), width (midpoint (r = 0.1344, *P*<0.0001) and maximum (r = 0.1316, *P*<0.0001).) The TA shape designations (Circular, Rectangular, and Ellipsoid) were significantly associated with most of the remaining traits evaluated. Pericarp thickness was negatively correlated for TA Circular (r = -0.2899, *P* < 0.0001) and positively correlated with Rectangular (r = 0.1347, *P* <0.0001) categories, indicating that pericarp was thicker for more rectangular peppers and less thick for circular peppers.

Perimeter was positively correlated with TA calculated categories Ellipsoid (r = 0.4086, *P*< 0.0001) and Circular (r = 0.3203, *P*<0.0001) categories, with moderate r values. Fruit shape index was positively correlated with the TA Circular (r = 0.8434, *P*< 0.0001) and Ellipsoid (r = 0.3052, *P* < 0.0001) and negatively correlated with TA Rectangular (r = -0.2225, *P*< 0.0001). Based on correlations among fruit traits, only fruit shape index, pericarp thickness, fruit shape triangle, and shoulder height were used for disease-fruit trait correlations.

Previously, a small, yet significant, isolate-specific correlation between fruit shape and disease susceptibility to *P*. *capsici* in a small mapping population was identified [40]. However, no other fruit traits were correlated with disease susceptibility. In a study by Biles et al, cuticle thickness, but not pericarp thickness was associated with disease resistance [43]. In this study, susceptibility to Phytophthora fruit rot was significantly positively correlated with pericarp thickness for both isolates evaluated at three and five dpi (Table 7). Fruit shape was negatively correlated with isolate OP97 at five dpi (r = -0.1618, *P*<0.0001), and isolate 12889 at three (r = -0.0684, *P* = 0.0362) and five (r = -0.1221, *P* = 0.0002) dpi. Correlations were significant, albeit weak, suggesting that fruit shape and thickness may be linked to disease susceptibility in some genetic backgrounds. Fruit shape triangle was weakly positively associated with susceptibility to isolate 12889 at five dpi (r = 0.0682, *P* = 0.0368). Shoulder height was positively associated with susceptibility to isolate 12889 at three (r = 0.0953, *P* = 0.0035) and five (r = 0.0847, *P* = 0.0094) dpi. Fruit shape triangle and shoulder height were not significantly correlated with susceptibility to isolate OP97. These correlations were both weaker and isolate specific, suggesting that breeders will be able to separate the traits with minimal effort. Understanding the broad sense heritability and potential correlations of fruit traits can greatly reduce the time to develop a commercially acceptable cultivar. In the Solanaceae, wild relatives are an important source of important horticultural traits such as abiotic and biotic resistance [21,22,44,45]. When these traits are incorporated into commercial backgrounds, deleterious or undesirable characteristics must be removed through repeated backcrossing to a commercial parent. This can take numerous generations depending on the trait, its ease of phenotyping, heritability, and any available molecular markers. In some instances, these traits may also be negatively linked with favorable traits such as yield or disease susceptibility [23,25].

**Table 7:** Pearson’s correlation coefficient (r) between pepper fruit traits and disease resistance to *Phytophthora capsici*.

Category	Pericarp	FSI^A^	FST^B^	SH^C^
**OP Day 3^D^**	0.1179***	-0.0533	-0.0005	-0.0041
**OP Day 5**	0.2197***	-0.1618***	0.0295	0.0363
**128 Day 3**	0.1888***	-0.0684*	-0.0213	0.0953**
**128 Day 5**	0.1961***	-0.1221***	0.0682*	0.0847**

^A^ Fruit shape index 1

^B^ Fruit shape triangle

^C^ Shoulder height

^D^ Disease at 3 and 5 days post inoculation by *Phytophthora capsici* isolates OP97 (OP) and 12889 (128).

* indicates significant at P ≤ 0.05

** P ≤ 0.01, and

*** P ≤ 0.001.

## Conclusion

Previously, fruit shape was negatively correlated with disease resistance to a single isolate, but no correlation was detected with pericarp thickness in a pepper mapping population [15]. However in this study, disease susceptibility was positively correlated with increased pericarp thickness for both isolates at three and five dpi. Based on these results, thin fruit were more resistant to *Phytophthora capsici* across the collection. Fruit shape was also negatively correlated with disease susceptibility to both isolates at 5 dpi, consistent with the previous study [15]. Fruit perimeter was positively associated with disease susceptibility for isolate 12889 at three and five dpi, but not OP97. Similar results with fruit shape triangle and shoulder height suggest that isolate-specific correlations may also confound breeding for fruit traits.

These data suggest that peppers with thicker flesh, similar to those seen in North America and Europe, tend to be more susceptible to *P*. *capsici*. While this does not directly translate to a reduction in yield, it indicates that breeding thick-fruited bell peppers with the preferred size and shape and sufficient Phytophthora fruit rot resistance may be a challenge. Negative and positive correlations among fruit horticultural traits and disease resistance traits can complicate the breeding process. However, using controlled fruit characteristic vocabularies, and understanding the correlations among fruit traits and disease resistance will be essential for continued crop improvement.

## Supporting Information

S1 Table Pepper fruit measurements for width at midpoint (width at mid), height at midpoint (height at mid), fruit shape triangle, and ellipsoid in cm. (XLSX)

S2 Table Pearson Correlation Coefficients for fruit traits. (XLSX)
